# A Novel Chemical Profile of a Selective In Vitro Cholinergic Essential Oil from *Clinopodium taxifolium* (Kunth) Govaerts (Lamiaceae), a Native Andean Species of Ecuador

**DOI:** 10.3390/molecules26010045

**Published:** 2020-12-23

**Authors:** Sandra Espinosa, Nicole Bec, Christian Larroque, Jorge Ramírez, Barbara Sgorbini, Carlo Bicchi, Nixon Cumbicus, Gianluca Gilardoni

**Affiliations:** 1 Departamento de Química y Ciencias Exactas, Universidad Técnica Particular de Loja, Loja 1101608, Ecuador; etsandra1@gmail.com (S.E.); cjlarroque@gmail.com (C.L.); jyramirez@utpl.edu.ec (J.R.); 2 IRMB, Université de Montpellier, INSERM, 34298 Montpellier, France; nicole.bec@inserm.fr; 3 Supportive Care Unit, Institut du Cancer de Montpellier (ICM), 34298 Montpellier, France; 4 Dipartimento di Scienza e Tecnologia del Farmaco, Università degli Studi di Torino, 10125 Torino, Italy; barbara.sgorbini@unito.it (B.S.); carlo.bicchi@unito.it (C.B.); 5 Departamento de Ciencias Biológicas, Universidad Técnica Particular de Loja (UTPL), Loja 1101608, Ecuador; nlcumbicus@utpl.edu.ec

**Keywords:** *Clinopodium taxifolium*, *Gardoquia taxifolia*, *Satureja taxifolia*, essential oil, GC-MS, GC-FID, enantioselective analysis, AChE, BChE, Ecuador

## Abstract

A novel chemical profile essential oil, distilled from the aerial parts of *Clinopodium taxifolium* (Kunth) Govaerts (Lamiaceae), was analysed by Gas Chromatography-Mass Spectrometry (GC-MS, qualitative analysis) and Gas Chromatography with Flame Ionization Detector (GC-FID, quantitative analysis), with both polar and non-polar stationary phase columns. The chemical composition mostly consisted of sesquiterpenes and sesquiterpenoids (>70%), the main ones being (*E*)-*β*-caryophyllene (17.8%), *α*-copaene (10.5%), *β*-bourbonene (9.9%), *δ*-cadinene (6.6%), *cis*-cadina-1(6),4-diene (6.4%) and germacrene D (4.9%), with the non-polar column. The essential oil was then submitted to enantioselective GC analysis, with a diethyl-*tert*-butyldimethylsilyl-*β*-cyclodextrin diluted in PS-086 chiral selector, resulting in the following enantiomeric excesses for the chiral components: (1*R*,5*S*)-(−)-*α*-thujene (67.8%), (1*R*,5*R*)-(+)-*α*-pinene (85.5%), (1*S*,5*S*)-(−)-*β*-pinene (90.0%), (1*S*,5*S*)-(−)-sabinene (12.3%), (*S*)-(−)-limonene (88.1%), (*S*)-(+)-linalool (32.7%), (*R*)-(−)-terpinen-4-ol (9.3%), (*S*)-(−)-*α*-terpineol (71.2%) and (*S*)-(−)-germacrene D (89.0%). The inhibition activity against acetylcholinesterase (AChE) and butyrylcholinesterase (BChE) of *C. taxifolium* essential oil was then tested, resulting in selective activity against BChE with an IC_50_ value of 31.3 ± 3.0 μg/mL (positive control: donepezil, IC_50_ = 3.6 μg/mL).

## 1. Introduction

According to the UN Environment Program World Conservation Monitoring Centre [1], Ecuador belongs to the group of the 17 megadiverse countries in the world, making it an extraordinary reservoir of biodiversity. As a megadiverse country, it must possess “at least 5000 of the world’s plants as endemics” [1]. Most of the botanical species described in Ecuador have never been studied so far from a phytochemical point of view; this makes the country an unbelievable source of potentially new chemical structures and biologically active compounds. In 2016, two of us (J.R. and G.G.) reported that about 50% of all the phytochemical publications on the Ecuadorian flora referred only to 8 of the 245 botanical families of the native species known in the country [2]. For this reason, the authors have been involved for years in the description of specialized (secondary) metabolites contained in the Ecuadorian flora, to contribute to the advance in its phytochemical and phytopharmaceutical knowledge [3,4,5,6,7,8,9,10]. Within a project focused on the description of new essential oils (EOs) [11,12,13,14,15,16,17,18,19,20], the aim of this study is to describe a novel chemical profile of an already known EO, distilled from the aerial parts of *Clinopodium taxifolium* (Kunth) Govaerts. *Clinopodium taxifolium* (Kunth) Govaerts is a species belonging to the family Lamiaceae, also known with many synonyms, such as *Gardoquia taxifolia* Kunth, *Satureja andrei* Epling, *Satureja taxifolia* (Kunth) Briq., *Gardoquia glabrata* Kunth, *Thymus taxifolius* Willd. ex Benth, *Satureja glabrata* (Kunth) Briq., *Satureja taxifolia* (Kunth) Briq. and *Satureja lineata* Epling. [10,21,22,23]. This plant is also known with the homonym *Clinopodium taxifolium* (Kunth) Harley [21]. The species is native of the Andean region of Ecuador [23], where it has been described in the provinces of Azuay, Oro y Loja. However, specimens have also been described in Bolivia and Peru [21]. This plant grows at an altitude of 1500–3000 m above sea level [23] and it is known as *Culantrillo de Cerro* or *Polea de Castilla* [10]. Furthermore, *C. taxifolium* is used by infusion in folk medicine for treating internal inflammations, flatulence, stomach pain, malaria and cough [10]. In 2018, one of the authors (G.G.) first published the composition of an EO from this species as a part of a phytochemical study [10]. Afterwards, the authors studied the composition of the EO of a different sample of *C. taxifolium* of different origin, obtaining very different quali-quantitative and general sensory results, compared to that described in 2018. The EO was therefore re-investigated on a statistically significant number of samples, rigorously identified from the botanic point of view and submitted to new biological essays.

The inhibition activity of a cholinesterase (ChE) was here investigated, within a project focused on the identification of plant specialized metabolites active against Alzheimer’s disease (AD). AD is a chronic neurodegenerative illness, characterized by a progressive deterioration of memory and cognitive functions. According to the World Health Organization (WHO), AD is currently the first cause of dementia in the world, being responsible for 60–70% of cases [24]. The universal interest in finding new anti-AD drugs is due to the rapid diffusion of this illness in western countries, mainly as a consequence of the increase in life expectancy. The 2016 World Alzheimer’s Report indicated that 47 million people live with dementia worldwide, and this number is expected to increase to more than 131 million by the year 2050. The disease is due to the accumulation of anomalous protein fragments (amyloid *β* peptides and hyper-phosphorylated tau proteins) into the brain. Several hypotheses try to explain this phenomenon, the most important of them being the so-called cholinergic hypothesis Accordingly, the cognitive degradation is due to the destruction of cholinergic neurons and the consequent ChE depletion, which can be counteracted by inhibiting the acetylcholinesterase (AChE) and butyrylcholinesterase (BChE) enzymes [24]. The administration of ChE inhibitors has been shown to produce an increase in the levels of acetylcholine (ACh) in the brain [25,26], counteracting the progress of the symptoms. The present discussion on the effectiveness and availability of drugs to treat AD [27,28], is the basis for the investigation of natural products in this field [26].

To the best of the authors’ knowledge, the present study is the first description of these chemical profile and biological activity of an EO distilled from *C. taxifolium*.

## 2. Results

### 2.1. Chemical Analysis

The EO was obtained in a quite low yield (0.07 ± 0.02%) from fresh plant material. A total of 37 compounds were detected, of which 32 with a non-polar Gas Chromatography (GC) column and 36 on a polar column. Most of the detected constituents were identified according to the corresponding Electron Ionization Mass Spectrum (EIMS) and Linear Retention Index (LRI). Two sesquiterpene hydrocarbons (204 amu) and one sesquiterpene alcohol (220 amu) could not be identified (unknown). The component abundance, here reported for both GC on non-polar (first value) and polar columns (second value), was measured by normalized percent abundance. The abundance of a total of 34 components was determined with at least one column, obtaining values corresponding to 88.1% and 86.0% of the whole sample, respectively, with a detection threshold fixed at 0.1%. The chemical analysis showed that this EO mainly consisted of sesquiterpenes and sesquiterpenoids (more than 70%), the main ones being (E)-*β*-caryophyllene (17.8–14.5%), *α*-copaene (10.5–8.0%), *β*-bourbonene (9.9–8.2%), *δ*-cadinene (6.6–5.4%), cis-cadina-1(6),4-diene (6.4–0.2%) and germacrene D (4.9–4.9%). The full chemical composition is reported in Table 1.

### 2.2. Enantioselective Analysis

**Figure 1:**
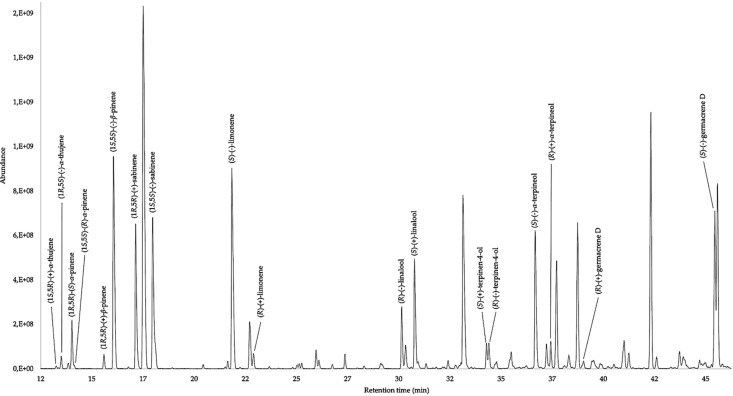
Enantioselective chromatogram of *C. taxifolium* EO on 30% diethyl-tert-butyldimethylsilyl-*β*-cyclodextrin/PS-086 column.

The enantioselective analysis was carried out on a 30% diethyl-*tert*-butyldimethylsilyl-*β*-cyclodextrin in a PS-086 capillary column. A total of nine chiral components were identified, eight of them were monoterpenoids and one a sesquiterpene hydrocarbon. None of the detected chiral compounds were enantiomerically pure. The results of the enantioselective analysis are reported in Table 2 and in Figure 1.

### 2.3. Cholinesterase Inhibition Test

**Figure 2:**
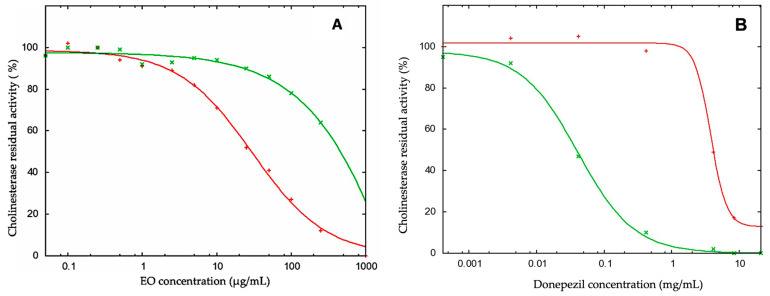
Half maximal inhibition concentration (IC_50_) of *C. taxifolium* essential oil (**A**) and donepezil (**B**) against AChE (green curve) and BChE (red curve).

The inhibitory activity of *C. taxifolium* EO against BChE showed an IC_50_ value of 31.3 ± 3.0 μg/mL; however, it was shown to be inactive against AChE (IC_50_ > 250 μg/mL) (Figure 2). These results were compared to the ones of donepezil as positive control, which showed an IC_50_ value of about one order of magnitude less against BChE.

## 3. Discussion

### 3.1. Selective BChE Inhibition Activity

The activities on the Central Nervous System (CNS) are not usually investigated for the EOs [39], due to antibacterial and antifungal properties being the most common. Nevertheless, the literature reports several volatile fractions with an interesting in vitro inhibition activity [24,40,41] versus AChE and BChE. Some of them are also significantly active against AD in vivo, or even in clinical trials [24]. The secondary metabolites that have been recognized to be active as pure compounds are linalool, *α*-terpinene, carvacrol, *α*-terpineol, thymol, *α*-pinene, (*E*)-*β*-caryophyllene and eugenol [24]. Five of them are also present in the investigated EO of *C. taxifolium*, where (*E*)-*β*-caryophyllene is the main constituent with an abundance of 17.8–14.5% on the two columns, respectively. However, the *C. taxifolium* EO produced a selective inhibition of BChE, differing from what is usually described in the literature, where (*E*)-*β*-caryophyllene, and some of the above mentioned terpenoids, inhibit both enzymes [41]. Actually, the selective inhibition of BChE is a very interesting property, since many studies have been investing the clinical application of similar drugs during the last 20 years [42,43,44]. AChE and BChE are both present in the CNS but in different locations. In particular, AChE is typically found in neurons, whereas BChE is common in glial cells [42]. In normal brain, about 80% of the total cholinesterase is constituted by AChE, and only 20% by BChE. However, with the progress of the AD, the ratio between the activities of BChE and AChE can increase from the normal value of 0.5 up to 11 [42]. This phenomenon could convert BChE to the main ChE in the development of AD. From a speculative point of view, the inhibition mechanism for *C. taxifolium* EO can be explained considering the different shapes of the active sites. According to X-ray diffraction data, the selective activity can be achieved by using the additional space present in the active site of BChE [42], which implies that selective inhibitors are usually bigger molecules than those of AChE. Since this is not the case for *C. taxifolium* EO components, the most probable hypothesis is a synergic effect of the whole mixture, where the combined effect of many constituents is different from that of the sum of the same pure components. This behaviour has already been demonstrated for some essential oils and described in the literature, e.g., for *Salvia lavandulifolia* and *Melaleuca alternifolia* (tea tree) EOs [45,46]. The other major EO constituents were *α*-copaene (10.5–8.0%), *β*-bourbonene (9.9–8.2%), *δ*-cadinene (6.6–5.4%), *cis*-cadina-1(6),4-diene (6.4–6.2%) and germacrene D (4.9–4.9%). These components can be responsible for the selective synergic effect, although, to the best of the authors’ knowledge, no evidence has been reported in this respect. Although the IC_50_ value calculated for this EO is quite higher than the one calculated for the positive control, the EO can be considered an active product. In fact, donepezil (positive control) is a pure substance while the EO is a mixture of at least 37 compounds, most of them likely inactive.

### 3.2. Enantiomeric Abundance and Biological Activity

A second important factor influencing the biological activity of a complex mixture is the enantiomeric composition of its chiral components [47,48,49], which makes their recognition a step necessary for a correct definition of its chemistry and biological activity. The enantiomeric excess of the chiral compounds is also important to characterize another fundamental property of an EO, i.e., its olfactive profile [50]. The *C. taxifolium* EO was therefore analyzed by enantioselective GC-MS (Es-GC-MS), to determine the enantiomeric excess (*ee%*) of eight chiral monoterpenoids and one sesquiterpene hydrocarbon, resulting in the following *ee%* values: (1*R*,5*S*)-(−)-*α*-thujene (67.8%), (1*R*,5*R*)-(+)-*α*-pinene (85.5%), (1*S*,5*S*)-(−)-*β*-pinene (90.0%), (1*S*,5*S*)-(−)-sabinene (12.3%), (*S*)-(−)-limonene (88.1%), (*S*)-(+)-linalool (32.7%), (*R*)-(−)-terpinen-4-ol (9.3%), (*S*)-(−)-*α*-terpineol (71.2%) and (*S*)-(−)-germacrene D (89.0%).

### 3.3. Novel EO Chemical Profile in C. taxifolium

Regarding the different chemical profile, compared to the one previously described, most of the essential oils of the genus *Clinopodium* present in the literature can be divided into three main categories: (i) EOs based on oxygenated monoterpenoids, (ii) EOs based on sesquiterpenes and sesquiterpenoids, and (iii) EOs based on both classes of metabolites. The first group is absolutely dominant, and it can be considered typical for the composition of *Clinopodium* essential oils. It is characterized by a fresh and minty aroma, sometimes phenolic. Pulegone, isopulegone, piperitone, piperitenone, piperitenone oxide, menthone, isomenthone and menthol are the main constituents. 1,8-Cineole, menthofuran, carvacrol, thymol, linalool and their derivatives can also seldom be found [51,52,53,54,55,56,57,58,59,60,61,62,63,64,65]. The second group is decidedly less common, and includes species like *Clinopodium umbrosum* (M.Bieb.) Kuntze and *Clinopodium gracile* (Benth) Matsum, whose EO major components are (*E*)-*β*-caryophyllene, germacrene D, spathulenol, *β*-elemene, *α*-bergamotene and *cis*-*β*-farnesene, among others [66,67]. The third type of EO is very rare and it is characterized by the presence of major compounds of both monoterpene and sesquiterpene. This is the case for *Clinopodium chinense* (Benth.) Kuntze, whose volatile fraction contains, most prevalently, piperitone, (*E*)-*β*-caryophyllene and spathulenol [68]. According to this classification, the chemical composition of the volatile fraction described in this study clearly fits with the second EO profile, since (*E*)-*β*-caryophyllene (17.8–14.5%), *α*-copaene (10.5–8.0%), *β*-bourbonene (9.9–8.2%), *δ*-cadinene (6.6–5.4%), *cis*-cadina-1(6),4-diene (6.4–0.2%) and germacrene D (4.9–4.9%) are the most abundant constituents. Nevertheless, the EO described in 2018 for *C. taxifolium* belongs to the first group [10]. Therefore, two quite different chemical profiles of *C. taxifolium* EO can be hypothesized, with the one described here probably being rarer.

## 4. Materials and Methods

### 4.1. Materials and Equipment

The chemical analyses were run by gas chromatography-mass spectrometry (GC-MS), with a 6890N GC unit from Agilent Technologies, coupled with a quadrupole Mass Spectrometry Detector (MSD) 5973 (Santa Clara, CA, USA). The MSD was operated with an electronic ionization (70 eV) source, in scan mode, with a mass range detection of 35–350 *m*/*z*. The MS transfer line and ion source temperatures were 280 °C and 200 °C, respectively. The analyses were carried out with a non-polar DB-5ms capillary column (5%-phenyl-methylpolysiloxane, 30 m length, 0.25 mm internal diameter and 0.25 μm film thickness; J & W Scientific, Folsom, CA, USA) and a polar HP-INNOWax column (polyethylene glycol, 30 m length, 0.25 mm internal diameter and 0.25 μm film thickness; Agilent Technologies, Santa Clara, CA, USA).

Quantitative analyses were carried out with a 6890N GC-FID system from Agilent Technologies (Santa Clara, CA, USA), equipped with a 7683 autoinjector also from Agilent Technologies (Little Falls, DE, USA). Linear retention indices were calculated through a homologous series of linear alkanes, from *n*-nonane to *n*-pentacosane (C_9_ purity 99% from BDH, Dubai, UAE; C_10_–C_25_ purity 99% from Sigma-Aldrich, St. Louis, MO, USA).

The enantiomeric excesses of chiral components were determined with an enantioselective column based on 30% diethyl-*tert*-butydimethylsilyl-*β*-cyclodextrin diluted in PS-086. (length: 25 m, internal diameter: 0.25 mm, film thickness: 0.25 μm, from Mega, Legnano, Italy).

All the analytical grade (purity >99%) solvents, 5,5′-dithiobis-2-nitrobenzoic acid (DNTB), *Εlectrophorus electricus* acetylcholinesterase (Type VS, freeze-dried powder, 744 U/mg solid, 1272 U/mg protein), equine serum butyrylcholinesterase (lyophilized powder, 900 units/mg protein) and acetylthiocholine iodide were purchased from Sigma-Aldrich. A Varioskan Flash detection system was used for enzymatic inhibition experiments (Thermo Fisher Scientific, Waltham, MA, USA). Donepezil (purity >98%) was used as positive control of ChE inhibition (Sigma-Aldrich).

### 4.2. Plant Material

The aerial parts of *C. taxifolium* were collected on 24 May 2018 in the province of Loja, mount Villonaco, at an altitude of 2724 m above sea level. The geographic point was located at coordinates 004°00’00” S–079°17’00” W. In order to grant a statistically representative number of samples, five different specimens were collected within a radius of 500 m from these coordinates. The plant was collected with the permission of the Ministry of Environment of Ecuador (MAE-DNB-CN-2016-0048) and the specimens were identified by one of the authors (N.C.). A voucher specimen was also deposited inside the Universidad Técnica Particular de Loja herbarium (herbarium code: HUTPL), with the identification code VMZ_010. To ensure the correct botanical identification, both the current and the previous specimens (voucher n. PPN-la-101) were compared with an original sample from the herbarium of the Universidad Nacional de Loja (herbarium code: LOJA), with all showing morphologically identical results.

### 4.3. Isolation of the Essential Oil and Samples Preparation

Five analytical hydrodistillations were performed on the fresh aerial parts of each botanical specimen. These processes were carried out with a glass laboratory-scale Marcusson apparatus, with recycling of the lower phase. During each analytical distillation, seventeen grams of fresh plant material were hydrodistilled for 90 min and the essential oil collected in 500 μL of an extraction layer of cyclohexane, containing *n*-nonane as internal standard (0.7 mg/mL). The cyclohexane layers were recovered and directly injected for GC analyses.

After verifying the similarity of the five chemical patterns, the entire remaining plant material was gathered to perform a preparative distillation. For this purpose, 1.6 kg of fresh plant material were hydrodistilled for 4 h [69], with a stainless-steel Clevenger-type apparatus, obtaining a pure essential oil that separated spontaneously from the water phase.

All samples were stored in amber vials at −15 °C. The pure essential oil was used for biological tests, while the 5 laboratory-scale repetitions were used for chemical and enantioselective analyses.

### 4.4. Qualitative Chemical Analysis

The GC-MS analyses on DB-5ms were carried out under the following conditions: carrier gas: helium, constant flow rate: 1 mL/min; injection volume: 1 μL, injection mode: split (ratio of 40:1), injection temperature: 250 °C; temperature program: from 50 °C (1 min) to 250 °C (10 min) at 3 °C/min. Analyses on the HP-INNOWax column were carried out under the same conditions as for DB-5ms, only the final oven temperature was set at 230 °C.

The EO components were identified by comparing both their linear retention indices (LRIs), calculated according to Van Den Dool and Kratz [70], and their mass spectra to those reported in the literature and, where available, with authentic standards (see Table 1).

### 4.5. Abundance Chemical Analysis

The abundance analyses were carried out under the same instrumental conditions as those adopted above, with the exception of the temperature program: from 50 °C (1 min) to 180 °C at the rate of 3 °C/min, then 15 °C/min until 250 °C (15 min) for DB-5ms and 230 °C (15 min) for HP-INNOWax. FID conditions: hydrogen flowrate 30 mL/min, air flow 300 mL/min, temperature: 250 °C. The abundance composition was obtained by using relative response factors, calculated on the basis of the combustion enthalpy [71] and taking isopropyl caproate as a calibration standard. Isopropyl caproate was obtained by synthesis in one of the authors’ laboratory (G.G.) and its purity was 97% by GC. Furthermore, a calibration curve was constructed for each column. Six calibration standard dilutions were taken to build-up the calibration curves, corresponding to 0.6, 1.8, 4.3, 8.3, 16.8 and 34.3 mg of isopropyl caproate in 10 mL of cyclohexane, respectively. Nonane (7.0 mg) was used as internal standard. The calibration curves generated a correlation coefficient of 0.999 for both columns.

### 4.6. Enantioselective GC Analysis

The enantioselective GC-MS analysis was performed with a temperature program from 50 °C (5 min) to 220 °C (5 min) at 2 °C/min. The elution order was established by injecting, in the same instrumental conditions, mixtures of enantiomerically pure standards, available in one of the authors’ laboratory (C.B.).

### 4.7. Cholinesterase Inhibition Test

The activities against cholinesterase (ChE) were evaluated by a colorimetric protocol, adapted from Ellman et al. [72]. The catalyst efficiently hydrolyzes acetylthiocholine (ATCh), the sulphur analogue of the natural substrate of these enzymes. After hydrolysis, this substrate analogue produces acetate ion and thiocoline. Thiocoline, in the presence of the highly reactive dithiobisnitrobenzoate ion (DTNB), produces a yellow color, which can be monitored quantitatively by its spectrophotometric absorption at 412 nm. The inhibition assay volume contained 200 μL of phosphate buffered saline (pH 7.4), DNTB (1.5 mM) and test sample in DMSO (1% *v*/*v*). Both *Electrophorus electricus* acetylcholinesterase and equine serum butyrylcholinesterase were dissolved in PBS pH 7.4 and were used at 25 mU/mL for the assay. After 10 min of preincubation, the substrate acetylthiocholine iodide (1.5 mM) was added to start the reaction. During the incubation at 30 °C for 30 min, multiple 96-well microliter sites were read in a Varioskan Flash detection system. All measurements were run in triplicate. When possible, the IC_50_ values were calculated using the GNUPLOT package online (www.ic50.tk, www.gnuplot.info). Donepezil was used as reference ChE inhibitor, with an IC_50_ = 100 nM for AChE and 8500 nM for BChE. In this assay, the possibility of false positive inhibition results, previously described for high concentration (>100 μg/mL) of amine or aldehyde compounds, cannot be excluded [73]. 

## 5. Conclusions

The aerial parts of the native Andean species *Clinopodium taxifolium* (Kunth) Govaerts (Lamiaceae) give an EO mainly consisting of sesquiterpenes and sesquiterpenoids. Although rather uncommon, the composition based on sesquiterpenoids has already been described for other species belonging to *Clinopodium* genus. In this case, it indicates the existence of at least two chemical profiles for *C. taxifolium.* This EO is characterized by a selective inhibition activity versus the enzyme BChE. The interest in a selective inhibitor is justified by the relative overexpression of this enzyme in the advanced progression of the AD. The search for this kind of inhibitor, over the last 20 years, testifies to the importance of this matter in pharmaceutical research. However, in our case, the relatively small size and the aliphatic structure of the molecules suggest a possible synergic mechanism instead of the presence of a single inhibitor in the mixture. This problem is possibly the main item to be investigated for this EO in future research.

## Figures and Tables

**Table 1:** Chemical composition of *C. taxifolium* EO with 5%-phenyl-methylpolysiloxane (DB-5ms) and polyethylene glycol (INNOWax) columns.

N°	Component	DB-5ms				HP-INNOWax			
		LRI ^a^	LRI ^b^	% ^c^	σ ^d^	LRI ^a^	LRI ^b^	% ^c^	σ ^d^
1	*α*-thujene	919	924 [29]	trace	-	1022	1025 [30]	trace	-
2	*α*-pinene	925	932 [29]	0.7	0.22	1017	1020 [31]	0.4	0.19
3	sabinene	965	969 [29]	3.2	1.67	1119	1122 [32]	3.0	1.42
4	*β*-pinene	968	974 [29]	3.5	1.37	1106	1105 [30]	2.9	1.06
5	*α*-terpinene	1012	1014 [29]	trace	-	1176	1178 [32]	trace	-
6	limonene	1024	1024 [29]	2.6	2.91	1198	1198 [32]	1.8	2.68
7	1,8-cineole	1026	1026 [29]	1.3	1.92	1205	1211 [32]	1.2	2.04
8	terpinolene	1078	1086 [29]	trace	-	1280	1282 [32]	trace	-
9	linalool	1101	1095 [29]	0.5	0.59	1554	1543 [32]	0.6	0.61
10	1-ethenyl-4-methoxy-benzene	1147	1154 [33]	1.6	0.04	1679	1670 [34]	0.3	0.02
11	citronellal	1151	1148 [29]	1.0	0.02	1448	1469 ^e^	0.8	0.02
12	*cis*-pinocamphone	1167	1172 [29]	0.8	1.09	1537	1545 [32]	1.2	1.08
13	Terpinen-4-ol	1174	1174 [29]	0.6	0.41	1600	1601 [32]	0.2	0.35
14	*α*-terpineol	1191	1186 [29]	0.3	0.30	1672	1694 [32]	0.3	0.04
15	methyl geranate	1320	1322 [29]	1.0	0.03	-	-	-	-
16	*α*-copaene	1363	1374 [29]	10.5	0.36	1483	1491 [32]	8.0	0.38
17	*β*-bourbonene	1369	1387 [29]	9.9	0.32	1509	1523 [31]	8.2	0.36
18	(*Z*)-*β*-caryophyllene	1389	1408 [29]	2.7	0.06	1565	1588 [32]	0.9	0.14
19	(*E*)-*β*-caryophyllene	1403	1417 [29]	17.8	1.26	1587	1599 [32]	14.5	1.30
20	*β*-cubebene	1414	1387 [29]	2.0	0.03	1580	1580 [35]	0.6	0.04
21	*α*-humulene	1438	1452 [29]	0.9	0.08	1658	1667 [32]	1.2	0.07
22	*cis*-cadina-1(6),4-diene	1466	1461 [29]	6.4	0.41	1664	-	6.2	0.05
23	germacrene D	1480	1480 [29]	4.9	0.65	1669	1674 ^e^	4.9	0.72
24	*δ*-cadinene	1506	1522 [29]	6.6	0.28	1751	1756 [32]	5.4	0.47
25	elemol	1539	1548 [29]	2.1	0.78	2079	2079 [32]	1.5	1.75
26	hedycaryol	1542	1546 [29]	0.1	0.10	2046	2037 [36]	1.2	0.48
27	spathulenol	1562	1577 [29]	0.9	0.33	2119	2121 [32]	1.4	1.60
28	10-epi-*γ*-eudesmol	1618	1622 [29]	1.8	0.51	2164	2170 [31]	1.3	0.24
29	caryophylla-4(12),8(13)-dien-5*β*-ol	1621	1639 [29]	0.4	0.62	2292	2299 [37]	1.6	0.54
30	*β*-eudesmol	1638	1652 [29]	1.0	1.11	2222	2220 [31]	1.1	0.31
31	*γ*-eudesmol	1639	1630 [29]	1.7	0.37	-	-	-	-
32	unknown (mw = 204)	-	-	-	-	1697	-	2.1	0.36
33	bicyclogermacrene	-	-	-	-	1723	1735 [32]	2.6	1.02
34	caryophyllene oxide	-	-	-	-	1968	1970 [31]	3.0	0.42
35	unknown (mw = 220)	-	-	-	-	2144	-	2.1	0.66
36	*α*-eudesmol	1651	1652 [29]	1.3	0.30	2214	2223 [32]	1.3	0.31
37	unknown (mw = 204)	-	-	-	-	2247	-	4.2	2.21
	*Monoterpene hydrocarbons*			*10.0*				*8.1*	
	*Oxygenated monoterpene*			*5.5*				*4.3*	
	*Sesquiterpene hydrocarbons*			*61.7*				*58.8*	
	*Oxygenated sesquiterpene*			*9.3*				*14.5*	
	*Others*			*1.6*				*0.3*	
	*Total amount*			*88.1*				*86.0*	

^a^Calculated linear retention index (LRI); ^b^ reference linear retention index; ^c^ content; ^d^ standard deviation; trace σ0.1%; mw = molecular weight; ^e^ identification confirmed by injection of original standards by one of the authors (B.S.).

**Table 2:** Enantioselective analysis of some chiral constituents of *C. taxifolium* EO on diethyl-*tert*-butyldimethylsilyl-*β*-cyclodextrin column.

Component	RT ^a^ (min.)	LRI ^b^	Enantiomer Percentage	*ee*%
(1*S*,5*R*)-(+)-*α*-thujene ^c^	12.96	920	16.1	67.8
(1*R*,5*S*)-(−)-*α*-thujene ^c^	13.20	924	83.9	
(1*R,*5*R*)-(+)-*α*-pinene	13.72	933	92.8	85.5
(1*S,*5*S*)-(−)-*α*-pinene	13.85	935	7.2	
(1*R*,5*R*)-(+)-*β*-pinene	15.28	959	5.0	90.0
(1*S*,5*S*)-(−)-*β*-pinene	15.76	967	94.9	
(1*R*,5*R*)-(+)-sabinene	16.85	985	43.9	12.3
(1*S*,5*S*)-(−)-sabinene	17.66	998	56.2	
(*S*)-(−)-limonene	21.55	1061	94.1	88.1
(*R*)-(+)-limonene	22.59	1078	5.9	
(*R*)-(−)-linalool	29.84	1198	33.7	32.7
(*S*)-(+)-linalool	30.47	1209	66.3	
(*S*)-(+)-terpinen-4-ol	33.98	1270	45.4	9.3
(*R*)-(−)-terpinen-4-ol	34.12	1272	54.6	
(*S*)-(−)-α-terpineol	36.37	1312	85.6	71.2
(*R*)-(+)-α-terpineol	37.13	1326	14.4	
(*R*)-(+)-germacrene D	38.74	1354	5.5	89.0
(*S*)-(−)-germacrene D	45.15	1474	94.5	

^a^ Retention time (RT); ^b^ Linear Retention Index (LRI) calculated on the 30% diethyl-*tert*-butyldimethylsilyl-*β*-cyclodextrin in PS-086 column; ^c^ tentative enantiomer identification according to [38]; *ee* = enantiomeric excess.

## Data Availability

The data presented in this study are available on request from the authors.
